# Brucellosis Seropositivity Using Three Serological Tests and Associated Risk Factors in Abattoir Workers in Gauteng Province, South Africa

**DOI:** 10.3390/pathogens13010064

**Published:** 2024-01-09

**Authors:** Francis B. Kolo, Abiodun A. Adesiyun, Folorunso O. Fasina, Bernice N. Harris, Jennifer Rossouw, Charles Byaruhanga, Hermanus De Wet Geyer, Lucille Blumberg, John Frean, Henriette van Heerden

**Affiliations:** 1 Faculty of Veterinary Science, Department of Veterinary Tropical Diseases, University of Pretoria, Pretoria 0182, South Africa; daydupe2003@yahoo.co (F.O.F.); cbyaruhanga27@yahoo.com (C.B.); henriette.vanheerden@up.ac.za (H.v.H.); 2 Faculty of Veterinary Science, Department of Production Animal Studies, University of Pretoria, Pretoria 0182, South Africa; aadesiyun@gmail.com; 3 School of Health Systems and Public Health, Faculty of Health Science, University of Pretoria, Pretoria 0084, South Africa; bernice.harris@up.ac.za; 4 Centre for Emerging, Zoonotic and Parasitic Diseases, National Institute for Communicable Diseases, a Division of the National Health Laboratory Service, Johannesburg 2132, South Africa; jennyr@nicd.ac.za (J.R.); hermang@nicd.ac.za (H.D.W.G.); lucilleb@nicd.ac.za (L.B.); johnf@nicd.ac.za (J.F.); 5 Faculty of Health Sciences, University of Witwatersrand, Johannesburg 2050, South Africa

**Keywords:** brucellosis, human, serology, abattoir, South Africa

## Abstract

Abattoir workers are liable to zoonotic infections from animals and animal products, primarily to diseases with asymptomatic and chronic clinical manifestations in animals, such as brucellosis. No published reports exist on the seroprevalence of brucellosis in abattoir workers in South Africa. Therefore, this cross-sectional study was conducted to estimate the occurrence and risk factors for *Brucella* exposure in abattoir workers in Gauteng Province. A total of 103 abattoir workers and managers from 6 abattoirs, where brucellosis-positive slaughtered cattle and sheep were previously detected, were interviewed and tested with serological assays using the Rose Bengal test (RBT), BrucellaCapt, and IgG-ELISA. A pre-tested questionnaire was administered to consenting respondents to obtain information on risk factors for brucellosis. Of the 103 respondents tested, the distribution of female and male workers was 16 (15.5%) and 87 (84.5%), respectively. The seroprevalence for exposure to brucellosis was 21/103 (20.4%, 95%CI: 13.1–29.5) using a combination of RBT, BrucellaCapt, or IgG-ELISA. For test-specific results, seroprevalences by RBT, BrucellaCapt, and IgG-ELISA were 13/103 (12.6%, 95%CI: 6.9–20.6), 9/103 (8.74%, 95%CI: 4.1–15.9), and 18/103 (17.5%, 95%CI: 10.7–26.2), respectively. Low-throughput abattoirs were identified as associated risks, as 29.3% of workers were seropositive compared with 12.7% of workers in high-throughput abattoirs, which highlights that direct contact at abattoirs poses higher risk to workers than indirect and direct contact outside abattoirs. This study confirms the occurrence of *Brucella* spp. antibodies among abattoir workers in South Africa, possibly due to occupational exposure to *Brucella* spp., and highlights the occupational hazard to workers. Furthermore, findings underscore that abattoir facilities can serve as points for active and passive surveillance for indicators of diseases of public health importance. We recommend periodic implementation of brucellosis testing of abattoir workers country-wide to establish baseline data for informing appropriate preventive practices and reducing the potential burden of infection rates among these high-risk workers.

## 1. Introduction

It has been established that, of all the 1415 known human infectious pathogens, 61% are zoonotic; 75% of emerging or re-emerging diseases are also thought to be zoonotic [1,2,3]. Similarly, 35% and 61% of emerging zoonotic pathogens are known to be transmitted through direct and indirect contact, respectively [1,2,3]. Apart from abattoirs being facilities where apparently healthy animals are slaughtered to provide wholesome meat for human consumption, abattoirs can also be used to monitor the effectiveness of disease control programs and disease surveillance [4,5,6,7]. However, zoonotic diseases pose an occupational hazard to abattoir workers, as they will have contact with subclinical infected animals, in which signs are either absent or too mild to be diagnosed. Such animals and their products pose a higher risk to abattoir workers, as they will not be aware of the pathogens and will have a greater chance of becoming infected.

Brucellosis is one of the neglected zoonotic diseases. It is endemic in most developing countries, with different clinical manifestations, including subclinical, acute, and chronic infections in animals [8]. *Brucella* spp. that infect farm animals include *B. abortus* (cattle), *B. melitensis* (sheep and goats), *B. suis* (pigs)*,* and *B. ovis* (sheep) [9,10]. *B. abortus* and *B*. *melitensis* cause infections in humans as undulant fever and Malta fever, respectively. The host preferences for *B. canis* and *B. suis* are dogs and pigs, respectively, and these species can cause brucellosis in humans [11,12,13,14].

It is an important zoonosis that infects humans, and about half-a-million humans worldwide are believed to be infected annually [12,15,16]. However, another study approximated global annual human incidence to be between 1.6 and 2.1 million [17]. Brucellosis causes an immense disease burden on infected individuals in terms of high socioeconomic and financial impact [8,17,18]. This impact is compounded by the fact that the clinical duration of human brucellosis varies from months to years and the prolonged episodes of clinical illness cause additional losses of time and money owing to disability [17,19,20,21]. The risk of brucellosis as an occupational hazard is relatively high among workers in abattoir facilities, meat packaging industries, and farms [22].

Brucellosis is endemic in the Mediterranean basin, the Caribbean, Africa, South and Central America, Asia, and the Middle East [23,24,25,26]. In these areas, *Brucella*-infected animals and their products slaughtered at abattoir facilities can serve as a source of infection for abattoir workers. Workers may be exposed to infection through direct contact with infected animals’ secretions or blood and aerosol dispersion of *Brucella*-containing droplets through inhalation or contact with the conjunctiva, mucous membranes, or compromised skin, especially for individuals without personal protective equipment (PPE) [24,25,27]. The abattoir facilities in South Africa adhere to enforced sanitary regulations in accordance with the Meat Safety Act 40 of 2000.

Brucellosis is a complex disease and humans can also become infected indirectly by consuming raw or undercooked meat (inadequately matured meat) or raw and unpasteurized milk or milk products [25,28]. A wide variety of risk factors that predispose abattoir workers to infection have been identified in studies and include length of days at work; unknown health status of slaughtered animals; non-use of PPE; splash of animal secretion on the face; hand-cut injuries during work; lack of hygienic measures in the work environment; and poor knowledge and attitude of the workers and their perception of zoonoses as occupational hazards [25,29,30,31,32,33,34,35,36]. These studies have been conducted in various countries/regions, each with its unique zoonotic disease profile in abattoir facilities that determined the risk factors identified in each study. In South Africa, the only published record focusing on occupational workers exposed to brucellosis is the seroprevalence amongst farm workers exposed to brucellosis case farms that were higher, ranging from 4.0% (BrucellaCapt^®^) to 16.7% (IgG ELISA^®^), compared with those exposed to brucellosis negative control farms where seroprevalence ranged from 1.9% (BrucellaCapt^®^) to 5.7% (IgG ELISA^®^) [37]. There are no empirical data or published reports on brucellosis seroprevalence and risk factors in abattoir workers in South Africa. 

The first proof of *B. abortus* infection in South Africa was in the year 1913, when contagious abortion in cattle was observed to spread across the country [38]. Initially, in the 1920s, only *B. melitensis* was isolated worldwide, and was suspected to be the cause of “camp fever” or Malta fever in South Africa. After the implementation of the bovine brucellosis scheme in 1979 [39], there has been limited information, with an increase in brucellosis prevalences over time, such as 1.5% brucellosis prevalence among slaughter cattle observed in an abattoir in the KwaZulu Natal Province that was recorded over three decades ago in the country [40]. In 2017, in the Eastern Cape Province, a 9.2% prevalence of *B. abortus* (of which 0.8% was the *B. abortus* S19 vaccine strain) was isolated from cattle, 2.9% *B. melitensis* from sheep, and 6.3% *B. melitensis* from goats using different sample types (blood, milk, and lymph nodes), followed by species confirmation using PCR [41]. Two other studies estimated bovine brucellosis prevalence in Gauteng that ranged from 1.79% to 5.5% from 2007 to 2019 [42,43]. The increase in animal brucellosis in South Africa suggests that humans are at greater risk to acquire brucellosis through contact with infected animals or their products.

Smits, et al. [44] indicated that immunoglobulin M (IgM) antibodies against human brucellosis develop early in the infection and remain for several weeks or months. Later, IgG antibodies develop and may be present at lower levels for months to years after the patient recovers. IgG but not IgM antibodies are present in recurring infections [44]. Thus, various serological tests are used to detect human brucellosis. Díaz, et al. [45] encouraged the use of the Rose Bengal test (RBT), as it is a rapid affordable test adaptable to serum dilutions that detects IgM, IgG, and IgA in short/acute and long/chronic brucellosis cases. RBT is highly specific in humans with no contact with *Brucella* and is comparable with the BrucellaCapt test for detecting IgM, IgG, and IgA. BrucellaCapt is an immunocapture assay recommended to detect the relapse of brucellosis in chronic/long cases [46]. IgG ELISA is an extremely sensitive serological test for detecting IgG antibodies found in long/acute cases [28]. This study was therefore conducted to obtain evidence-based data on the seroprevalence using RBT, BrucellaCapt, and IgG ELISA serological tests and associated risk factors for brucellosis in abattoir workers in Gauteng Province, South Africa.

## 2. Materials and Methods

### 2.1. Study Area

The study area was Gauteng Province of South Africa. The estimated number of livestock in Gauteng Province as of May 2018 was 246,395 cattle, 92,160 sheep, 29,017 goats, and 156,264 pigs [47], mostly belonging to commercial farmers. 

### 2.2. Study Design

A cross-sectional study was conducted to estimate the seroprevalence of *Brucella* spp. exposure using three serological tests (RBT, BrucellaCapt, *Brucella* ELISA IgG) among abattoir workers in selected abattoirs in Gauteng Province from 2017 to 2018. Six of the fourteen abattoirs from a previous study (where slaughtered livestock (cattle and sheep) were confirmed positive for brucellosis (*B. abortus* and *B. melitensis*) [42]) were included in the current study. Three of these six abattoirs were high-throughput facilities (slaughtering more than 20 cattle per day), while the other three were low-throughput facilities (slaughtering 20 or less cattle per day), and all processed multiple species of animals. 

### 2.3. Sampling Method and Recruitment

This study followed a willing participant approach. A total of 103 abattoir workers, managers, and meat inspectors consented to participate in this study. All abattoir workers/managers who met the eligibility criteria (those who were present and consented on the day of sampling) were interviewed. Respondents were classified by the type of duties they performed at the facilities, including butcher (slaughterman/offal cleaners) and others (inspector, transporter, and those engaged in any other type of work conducted at the abattoir). A questionnaire that elicited demographic data and duties performed at the abattoir was administered to each participant, and, when necessary, interpreters who were members of the research team were used. 

### 2.4. Study Population

The inclusion criteria for the study population were: being active in the meat industry and working in the abattoir industry. These included the managers and abattoir workers who submitted signed written consent forms. All abattoir managers who signed the consent forms as stakeholders permitted the team to conduct this study at their facilities. 

### 2.5. Sample Size of Abattoir Workers

The sample size for human workers in the abattoirs was determined according to Naing, et al. [48]: n_o_ = t^2^ (p) (1 − p)/d^2^, where t = 1.96, p = prevalence, d = precision at a type 1 error of 0.05, and n_o_ = estimated sample size. Since there are no current documented data on the seroprevalence of human brucellosis in abattoir workers in Gauteng Province or South Africa, the prevalence of 50% (0.5) was assumed. For this study, the minimum sample size was determined using the following criteria: *p* = 0.5 and a precision of 10%. For this study, a minimum sample size of 96 was adopted and a total of 103 humans was sampled based on 7 other workers volunteering to participate and who were available on the allocated day for testing. 

### 2.6. Pre-Testing of the Questionnaire and Data Collection

A structured questionnaire was constructed with three parts on Microsoft Word Office 2007. This was tested for clarity among five abattoir workers, and content was adjusted based on the responses obtained. The first part of the questionnaire extracted demographic data on the worker comprising the age, sex, number of years at work, job description, and the section where the interviewee was stationed at the slaughter facility. The second part consisted of close-ended questions about attitudinal risky practices during work, such as the use of protective equipment (PPE), eating raw meat, consumption of raw milk, and splash exposure of blood or fluid in the eyes or mouth during work. The last part consisted of questions regarding the subject’s perception of the probability of contracting zoonoses at the abattoir and their attitude towards seeking medical assistance whenever they felt any symptoms of illness.

### 2.7. Data Collection

All consenting abattoir workers who met the eligibility criteria were interviewed using the structured questionnaire. 

### 2.8. Collection of Blood Samples

A qualified phlebotomist was recruited to collect a maximum of 5 mL of venous blood from each consenting participant following the established procedure during and post-collection of blood samples. A total of 103 blood samples were collected in vacutainer tubes without any anticoagulant to harvest sera and stored under 4 °C prior to usage for the serological assays. 

### 2.9. Serological Tests Conducted 

All the serological tests were conducted in parallel and series at the Special Bacterial Pathogens Reference Laboratory of the National Institute for Communicable Diseases (NICD), South Africa. In parallel testing, two or more tests are applied. If any of the test results are positive, then the result is considered to be positive. In series testing, the tests are performed sequentially, but only positive results to the initial test are retested. This resulted in this study being based on parallel testing. 

Rose Bengal test (RBT): The commercial IDEXX *Brucella* antigen (Switzerland) stained with Rose Bengal (30 µL) was mixed with an equal volume of serum sample and the mixture was agitated gently for four minutes at room temperature on a shaker. Standard positive and negative controls supplied with the kit were treated according to the manufacturer’s protocol. Agglutination was read after four minutes, and any visible agglutination was regarded as positive for *Brucella* antibodies. The diagnostic sensitivity and specificity for the RBT used was assumed to be 100% and 75%, respectively, based on previous validation studies [49].

BRUCELLACAPT^®^ (Vircell S. L., Granada, Spain): Using the manufacturer’s instructions, this single-step immunocapture assay that detects non-agglutinating IgG and IgA and agglutinating antibodies [50] was performed, inclusive of the standard positive and negative controls. The reference of sensitivity and specificity of the BrucellaCapt was stated as 80.4% and 96.8%, respectively [50].

*BRUCELLA* ELISA IgG (Vircell S. L., Granada, Spain): This enzyme-linked immunosorbent assay (ELISA) was performed according to the manufacturer’s instructions. Human sera were added to microtiter plates with antigen adsorbed on the polystyrene surface. Unbound immunoglobulins were washed off, as the enzyme-labeled antihuman globulin bound the antigen–antibody complex in a second step. After another wash step, the bound conjugate was developed with the aid of a substrate solution (TMB) to render a blue-colored soluble product that turned yellow after adding an acid-stopping solution. The ELISA washer (ELx50/8), ELISA reader (ELx800), and software program used to read and interpret the results (GEN 5, version 3.08 Software package) were all BioTek (Winooski, VT, 0504-0998, USA) products. The positive and negative controls were supplied with the kit. The reference of sensitivity and specificity of the IgG-ELISA was 75.4% and 100%, respectively [50].

### 2.10. Statistical Analysis

Data were managed using Microsoft Excel sheet v 2016 (Microsoft Corporation, Redmond, WA, USA). All data were entered into the software and checked by two individuals independently, checking for consistency and errors in data entry. Each of the 17 variables was assessed by univariate analysis for association with *Brucella* positivity status (serology) using the chi-squared or Fisher’s exact test in two-by-two tables. The 17 variables were also assessed for collinearity using the Cramer’s V test. Collinearity was considered to be present if Cramer’s V was greater than 0.6. If two variables were collinear, only one more biologically plausible variable was selected for multivariable analysis. Other variables (*n* = 3) were excluded from multivariable analysis due to few participants in one or more categories or due to *p* > 0.15 from univariate analysis. Subsequently, only 3 of the 17 initial variables from univariate analysis were analyzed in binomial multivariable generalized linear models following a backward elimination approach. The goodness-of-fit of the multivariable models was performed and compared using the Hosmer–Lemeshow and Omnibus fit tests. Statistical analyses were conducted at 5% level of significance using the “MASS” and “rms” packages in R statistical software version 4.2.1 [51]. 

### 2.11. Ethical Approval

Research (V089-16) and human ethics approval (519/2017) was obtained from the University of Pretoria Faculty of Veterinary Sciences and Faculty of Health Sciences Research Ethics Committees. 

## 3. Results

### 3.1. Description of Participants

Of the 103 respondents evaluated, the distribution of females was 16/103 (15.5%) and 87/103 (84.5%) for males. The mean age of the respondents was 36.4 years (range 18–60). The mean age for females was 32.6 years (range 23–49), while for males it was 37.1 years (range 18–60). The distribution of respondents according to age was as follows: for 18-to 30-year-olds, it was 32/103 (31.1%), and for the 31-to 60-year-olds, it was 71/103 (68.9%). 

Based on the abattoir-related job specifications, the professionals interviewed were: slaughtermen (butchers) = 68/103 (66.0%); inspectors = 14/103 (14.0%); cleaners = 8/103 (8.0%); offal washers = 7/103 (7.0%); managers = 3/103 (3.0%); transporters = 2/103 (2.0%); and hide and skin processors = 1/103 (1.0%). The number of years worked at the abattoir were as follows: work duration of 1 year, 21/103 (20.3%); work duration of 2 years 17/103 (16.5%); and work duration of 3 years and above, 65/103 (63.1%). In addition, according to the number of days at work weekly: 98/103 (95.2%4) worked for 5 to 7 days per week, 2/103 (1.94%) worked for 3 to 4 days, and 3/103 (2.91%) worked for 1 to 2 days.

### 3.2. Analysis of Seropositivity by Different Tests among the Abattoir Workers

Of the 103 sera tested from the abattoir workers, 21 were seropositive on parallel testing (at least one of the serological tests), and indicated seroprevalence of 21/103 (20%, 95%CI: 13.1–29.5) for *Brucella* spp. exposure. Concurrent test results were as follows: with RBT, the number (percentage) of seropositive was 13 (12.6%, 95%CI: 6.89–20.6); with BrucellaCapt, this was 9 (8.74%, 95%CI: 4.07–15.9); and with IgG-ELISA assay, this was 18 (17.5%, 95%CI: 10.7–26.2) (Table 1).

In series, the number (percentage) seropositive with RBT and BrucellaCapt was 7 (6.80%, 95%CI: 2.78–13.5); with RBT and IgG-ELISA, this was 10 (9.71%, 95%CI: 4.75–17.1); with BrucellaCapt and IgG-ELISA, this was 9 (8.74%, 95%CI: 4.07–15.9); and with RBT, BrucellaCapt, and IgG-ELISA, this was 7 (6.80%, 95%CI: 2.78–13.5) (Table 1). 

### 3.3. Descriptive Statistics and Univariable Analysis of Factors among the Respondents Predictive of Brucella Seropositivity

A total of 17 variables were subjected to univariate analysis for association with *Brucella* seropositivity among abattoir workers (Table 2). The seropositivity on at least one test (RBT, and/or BrucellaCapt, and/or IgG ELISA) among female and male workers during this study period was 6.25% (1/16) and 22.9% (20/87), respectively, and was not statistically significant (*p* = 0.18) (Table 2). Among the age categories, seropositivity was higher (22.5%, 16/71) among older persons aged from 31 to 60 than younger workers aged from 18 to 30 (15.6%, 5/32), although the difference was not statistically significant (*p* = 0.42) (Table 2). The seropositivity among abattoir workers, stratified by the types of duties performed, was as follows: slaughtermen/offal washers (butchers/offal) was 22.7% (17/75) and others (inspectors, cleaners, transporters, managers, and hide and skin processors) was 14.3% (4/28); this was not statistically significant (*p* = 0.42) (Table 2). Assessment of other questionnaire responses showed that 8.3% of those who ate raw or undercooked meat were seropositive, while 23.7% of those without knowledge on brucellosis were seropositive to brucellosis; these were not statistically significant compared with their corresponding categories (Table 2). According to the types of abattoirs sampled, 29.3% of workers in low-throughput abattoirs were seropositive compared with 12.7% of workers in high-throughput abattoirs; this was statistically significant (*p* = 0.03) (Table 2).

All the 17 variables from univariate analyses were screened for inclusion in multivariable models. The following variables were excluded from the multivariable analysis due to few participants in one or more categories: days at work (98/103 participants worked for more than 1 day); negative previous brucellosis diagnosis (101/103 participants tested negative); regular PPE utilization (97/103 participants used PPE). One variable, “do you think you can get brucellosis from animals”, showed collinearity with knowledge about brucellosis (Cramer’s V = 0.87), but no collinearity was observed for other variables (Cramer’s V < 0.6), while 10 variables had *p*-value greater than 0.15 from univariate analysis. Therefore, of the initial 17 variables in Table 2 and Table 3 (abattoir, gender, and slaughter animals at home) were included in multivariable generalized linear models and, after a backward elimination procedure, data fit well the model that had only 2 variables (abattoir and gender) (Table 3) (Hosmer–Lemeshow test: χ^2^ = 0.013, df = 1, *p* = 0.91; omnibus test: Z = 0.54, *p* = 0.589). Workers from low-throughput abattoirs were more likely to be seropositive for *Brucella* spp. (OR = 5.3, *p* = 0.03) compared with those from high-throughput abattoirs, and exposure was higher among males (OR = 3.1, *p* = 0.12) than females (Table 3). 

## 4. Discussion

This research highlights the occupational hazard of diseases such as brucellosis with subclinical infections. Gauteng Province abattoir workers had moderate-to-low brucellosis seroprevalence, with 20% overall using a combination of serological tests (RBT, BrucellaCapt, and IgG ELISA) or 6.8–9.7% using series test combinations (Table 1). The current study was a continuation regarding abattoir workers after a previous report showed 5.5% brucellosis sero- (using RBT and iELISA) and culture-prevalences (*B. abortus* biovar 1, *B. melitensis* bv 2 and 3 from cattle tissues) in 200 bovines slaughtered in Gauteng Province. Most of the *Brucella* spp. isolated were from animals slaughtered at low-throughput abattoirs [42]. The associated risk to workers to brucellosis was influenced by the brucellosis infection in livestock with which the workers had contact. This was reflected by the low-to-moderate brucellosis seroprevalence (4.0% (BrucellaCapt) to 16.7% (IgG ELISA)) in farm workers on brucellosis-infected farms compared with low seroprevalence (1.9% (BrucellaCapt^®^) to 5.7% (IgG ELISA^®^)) on farms free of brucellosis in South Africa [37]. As mentioned, in South Africa, we have limited data on brucellosis prevalence in animals, which has increased despite the implementation of the bovine brucellosis scheme in 1979 that focused on vaccination, testing, and slaughtering of high-risk bovine (while leaving it voluntary for other livestock) [52], which highlighted the increased risk for humans. The studies mentioned earlier [42,43] reported brucellosis prevalence in a small percentage of livestock in the country, which was also similar to the case of humans (*n* = 103) tested in the current study. These studies provide a limited view of the prevalence of brucellosis in both livestock and humans in South Africa that needs to be expanded. However, these studies reflect the endemic nature of brucellosis in livestock in South Africa that poses a higher risk to humans in contact with infected animals. In addition to the human brucellosis seroprevalence reported in the current study, associated risk factors such as gender and abattoir type highlight that direct contact with animals and their products are important risk factors for abattoir workers rather than direct and indirect contact with animal products outside the abattoir facilities. However, some workers, who may also be farmers, could be infected elsewhere via the alimentary route. 

In humans, the clinical presentation of brucellosis can be ambiguous; as such, presumptive identification of infections is conducted based on morphologic, cultural, and serological properties [15]. Supportive evidence of human brucellosis diagnosis may include a demonstration of *Brucella* antigens in human blood using validated serological tests, as well as an indication of high sustained IgG antibody titers as seen in agglutination, complement fixation test, or ELISA with standardized antigens [25]. However, routine *Brucella* antigen detection tests are not regularly conducted in humans in South Africa. There is also no consensus on preferred serodiagnosis tests for human brucellosis because there is no single definitive test to detect the disease against which all other laboratory assays should be measured [53]. However, other diagnostic methods that can be used are cultures and PCRs to detect pathogen nucleic acid. Serological tests for brucellosis are frequently evaluated by comparing results to those obtained with other serological assays, used alone or in combination [53]. As mentioned, this in part is a result of the evolution of the disease either in the acute or chronic phase. IgM is present in acute cases and this immunoglobulin reverts to the background levels and IgG (IgA) dominates later in the chronic phase of the disease [45]. The immuno-response to brucellae is dominated by antibodies in the polysaccharide (PS) section of smooth (S) *Brucella* lipopolysaccharide (S-LPS), which elicits an IgM/IgG (IgA) shift [54]. Multiple tests for confirmation are also used to eliminate cross-reactivity of serological tests to other Gram-negative bacteria such as *E. coli* O:157, *Francisella tularensis,* and *Yersinia enterocolitica* 0:9 [55]. All this is considered based on the three serological tests used in this study to detect smooth *Brucella* antibodies in abattoir workers. For a reliable serological diagnosis of human brucellosis, at least two tests are required: one based on a high-sensitivity screening method and another based on more specific strategies to confirm the preliminary test results.

RBT is a rapid and sensitive screening test that determined 12.6% seropositivity in abattoir workers from this study. RBT can produce false positive results, especially in patients with cross-reactive organisms and healthy individuals who may have had contact with S-*Brucella* without developing the disease [25]. Screening RBT is recommended to be confirmed with other serological tests. In this study, RBT confirmed with IgG ELISA reported 9.7% seropositivity compared with 6.8% in RBT and BrucellaCapt (Table 1). We reported a lower seropositivity of 6.8% with RBT confirmed with both BrucellaCapt and IgG ELISA in series (Table 1). IgG ELISA (17.5%), as a parallel test, recorded higher seropositivity compared with both BrucellaCapt (8.7%) and RBT (12.6%) (Table 1). Further, the seropositivity (17.7%) recorded with parallel IgG ELISA was lowered (8.7%) when combined in series with BrucellaCapt. The evolution of immunoglobulin isotypes can be measured following infection and treatment using ELISA [25].

Xu et al. (2023) [56] evaluated BrucellaCapt and IgG ELISA in patients with suspect brucellosis and found that BrucellaCapt had excellent specificity (100%) and high positive predictive value (PPV) (100%), with sensitivity and negative predictive values of 88.3% and 86.3%, and found that the combination of BrucellaCapt and IgG ELISA had excellent diagnostic prediction. The current study determined that IgG ELISA plus BrucellaCapt had 8.7% seropositivity, which supported the evaluation of human brucellosis in an endemic area by six serological tests (RBT, standard serum agglutination (SAT), Brucella Coombs, BrucellaCapt, IgM ELISA, and IgG ELISA) that found all tests valuable for positive results, but only Brucella Coombs and BrucellaCapt were reliable for negative serological results [57]. Another study tested human brucellosis in an endemic area in Turkey [57] and reported that BrucellaCapt recorded higher seropositivity (74.0%) at 1/360 titer cut-off and 80.9% at titer cut-off value at 1/160 compared with other serological tests [57], thus suggesting at least two tests are needed [56,57]. From our results, the combination of an Ig ELISA and BrucellaCapt approach might be the most accurate reflection, with no false positive (Table 1). This work is the first abattoir worker-based study on human brucellosis to be conducted in South Africa, thereby providing relevant baseline data on this group. These findings also underscore the urgent need to replicate the study in other provinces in South Africa to fully understand the exposure status among the occupationally exposed groups in the country. This is imperative, because brucellosis is endemic in livestock in the country [41,42,58,59], and *B. melitensis* has been isolated and reported in humans [60]. 

Two abattoir workers indicated a previous diagnosis of the disease; one tested positive with RBT and BrucellaCapt, while the other tested negative with all tests. A study reported that the serological cure rate increased from 8.3% in the first 3 months to 71.4% after 2 years [61] as IgM became non-detectable and low levels of IgG occurred. The median time of serological cure was 18.5 months, and 28.6% of cured patients continued to have high titers for 2 years or more. Thus, the positive individual is plausible because brucellosis requires prolonged treatment, and the antibodies remain in the system for a very long time, even after treatment [25].

The risk factors associated with abattoir workers identified in this study, namely gender and abattoir type that only reflected direct contact with animals and products, were unexpected. Brucellosis is a complex disease and we thought that direct contact with animals, and especially indirect contact with animal products such as drinking raw milk, eating raw meat, and slaughtering at home (as reported in the literature [31,32,54,62,63]), would have been associated risk factors for these workers.

According to abattoir type, more workers from low-throughput abattoirs (29.2%) were seropositive for *Brucella* spp. compared with high-throughput abattoirs (12.7%) (*p*-value = 0.03). This is in agreement with previous findings involving 14 abattoirs in Gauteng Province, in which a higher prevalence was observed in cattle slaughtered in low-throughput abattoirs (22.4%) compared with those from high-throughput abattoirs (5.2%) [42]. It is an important finding, as one would expect that high-throughput abattoirs might have a higher risk, as low-throughput abattoirs slaughter a maximum of 20 animals per day, while high throughput abattoirs slaughter more than 20 (up to the maximum set by the capacity of the facility). However, other factors, such as better infrastructure, training, sanitation, and/or hygiene standards at high-throughput abattoirs could lead to a lower risk, which should be investigated. It cannot be over-emphasized that interventions need to be instituted to reduce the impact of identified risk factors as part of an overall objective to reduce the exposure potential and exposure experience of abattoir workers to brucellosis.

## 5. Conclusions

This study has determined, for the first time in South Africa, the occurrence of antibodies against *Brucella* spp. among abattoir workers, which could reflect current infection or previous exposure to *Brucella*. The risk factors associated with brucellosis seropositivity indicate that contact with animals and their products at abattoirs poses an occupational risk. We recommend implementing country-wide brucellosis testing of abattoir workers to establish baseline data that could indicate previous exposure, which could be used to mitigate and motivate appropriate preventive practices to reduce the infection rate among these high-risk workers. Finally, such an approach will also provide insight into the magnitude of infections by *Brucella* spp. among abattoir workers. The evidence-based data provided by our study provide baseline data for policymakers in decision making. The findings of this study underscore that abattoir facilities can be used for active and passive surveillance of some diseases of public health and economic importance.

## 6. Limitations of this Study

Isolation of the infecting *Brucella* spp. could not be conducted as respondents may not have been harbouring the bacteria at the time of testing but may only have had antibodies from previous exposure. Certain bacterial pathogens (*Yersinia enterocolitica* O:9, *Salmonella enterica* serovar Urbana O:30, *Francisella tularensis*, *Escherichia coli* O116 and O157, *Vibrio cholerae*, *Xanthomonas maltophilia* and *Afipia clevelandensis*) [9,64] that can cause false-positive serological test results for brucellosis due to cross-reactivity might have limited this serological study. Future studies should be conducted to isolate infectious *Brucellla* spp. and the evaluation of their pathogenicity. Our sample size may also be considered small, but it met the requirement for statistical evaluation. A nationwide analysis, utilizing a larger sample size, may present a more accurate result and comparison to the population. The number of female workers was small compared with male workers, hence this could have led to bias.

## Figures and Tables

**Table 1:** Overlap and non-overlap of brucellosis serological results for the Rose Bengal test (RBT), BrucellaCapt, and IgG-ELISA testing of Gauteng abattoir workers.

Test	No. of Sera Tested	No. (%) * Positive	95% CI *
RBT	103	13 (12.6)	6.89–20.62
BrucellaCapt	103	9 (8.7)	4.07–15.94
IgG-ELISA	103	18 (17.5)	10.70–26.21
RBT/BrucellaCapt	103	7 (6.8)	2.78–13.50
RBT/IgG-ELISA	103	10 (9.7)	4.75–17.13
BrucellaCapt/IgG-ELISA	103	9 (8.7)	4.07–15.94
RBT/BrucellaCapt/IgG-ELISA	103	7 (6.8)	2.78–13.50

* = %—percentage, CI—confidence interval.

**Table 2:** Descriptive statistics and univariate analyses for factors associated with *Brucella* seropositivity among abattoir workers in Gauteng abattoirs, South Africa.

Variable	Category	Number of Humans Sampled	Number Positive	Percentage of Seropositive	Odds Ratio (95% CI *)	*p*-Value
Abattoir type	High-throughput Low-throughput	55 48	7 14	12.7 29.2	2.79 (0.94, 9.11)	0.039
Gender	Female Male	16 87	1 20	6.3 23.0	4.44(0.61, 197.62)	0.183
Age category	18–30 years 31–60 years	32 71	5 16	15.6 22.5	1.56 (0.48, 6.05)	0.421
Marital status	Married Single	42 61	8 13	19.0 21.3	1.15 (0.39, 3.57)	0.779
Job description	Other Slaughterer/offal handling	28 75	4 17	14.3 22.7	1.75 (0.49, 7.89)	0.420
Days at work	1 to 4 days 5 to 7 days	4 99	2 19	0.5 19.2	0.24 (0.017, 3.53)	0.184
Years at work	More than 1 year Up to 1 year	82 21	16 5	19.5 23.8	1.29 (0.32, 4.44)	0.663
Working on farms other than abattoirs?	No Yes	65 38	16 5	24.6 13.2	0.47 (0.12, 1.51)	0.164
Do you consume unpasteurized milk?	No Yes	77 26	16 5	20.8 19.2	0.91 (0.23, 3.03)	0.865
Do you slaughter animals at home?	No Yes	54 49	8 13	14.8 26.5	2.06 (0.70, 6.41)	0.141
Do you eat raw or undercooked meat?	No Yes	91 12	20 1	22.0 8.3	0.33 (0.007, 2.49)	0.451
Do you always wear your PPE?	No Yes	6 97	0 21	0.0 21.6	Infinity	0.342
Do you know of brucellosis?	No Yes	76 27	18 3	23.7 11.1	0.41 (0.07, 1.59)	0.265
Do you think you can get brucellosis from animals?	No Yes	81 22	10 0	12.3 0	0.33 (0.03, 1.57)	0.231
Have you had cut/hand-cut injuries on duty?	No Yes	26 77	6 15	23.1 19.5	0.81 (0.25, 2.89)	0.694
Have you ever been diagnosed with brucellosis?	No Yes	101 2	20 1	19.8 50	3.98 (0.05, 320.83)	0.368
Have you ever had animal blood splashed on your face?	No Yes	23 80	3 18	13.0 22.5	1.92 (0.48,11.25)	0.393

* CI—confidence interval.

**Table 3:** Multivariable analysis.

Multivariable Analysis			
Variable	Category	Odds Ratio (95% CI *)	*p*-Value
Abattoir	High-throughput (ref)		
	Low-throughput	5.32 (0.64, 43.93)	0.03
Gender	Female (ref)		
	Male	3.11 (1.12, 8.67)	0.12

* CI—confidence Interval. * ref—reference.

## Data Availability

The data presented in this study are available on request from the corresponding author. The data are not publicly available due to privacy restrictions.
